# 1172. Compliance with Human Papillomavirus Vaccination At The HIV Outpatient Program in New Orleans, Louisiana

**DOI:** 10.1093/ofid/ofad500.1012

**Published:** 2023-11-27

**Authors:** Tat Yau, Maria Frontini, Michael Hagensee

**Affiliations:** LSU Health Sciences Center New Orleans, New Orleans, Louisiana; Louisiana State University Health Sciences Center, New Orleans, Louisiana; LSU Health Sciences Center New Orleans, New Orleans, Louisiana

## Abstract

**Background:**

Guidelines recommend the human papillomavirus (HPV) vaccine for all adults through age 26, shared clinical decision-making for those aged 27 through 45 years who are inadequately vaccinated, and for all people with HIV up to age 45 years. The intent of this study is to evaluate HPV vaccination rates at the HIV Outpatient Program (HOP) at University Medical Center in New Orleans.

**Methods:**

Immunization records from patients aged 18 through 45 years attending a physician visit in the LSU Infectious Diseases fellows’ HIV primary care clinic at HOP in 2022 were reviewed. The purpose of this project was to assess compliance with HPV vaccination recommendations among the infectious diseases fellows.

**Results:**

Among patients under age 27, 15 of 21 visits (71%) had completed HPV vaccines series. 4 visits (19%) had incomplete status and 2 visits (10%) did not have any HPV vaccines record. Among patients aged 27 through 45 years, 73 of 194 visits (38%) had completed HPV vaccines series. 34 visits (18%) had partial vaccination records and 87 visits (45%) had no records.

**Figure:**
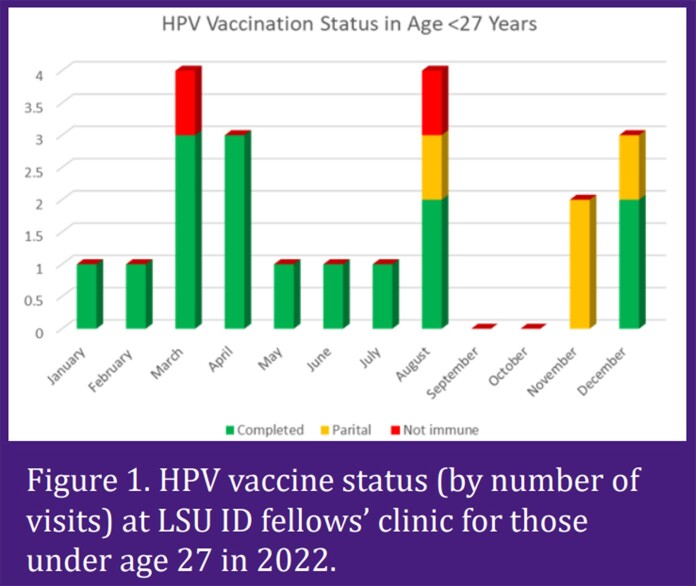

**Figure:**
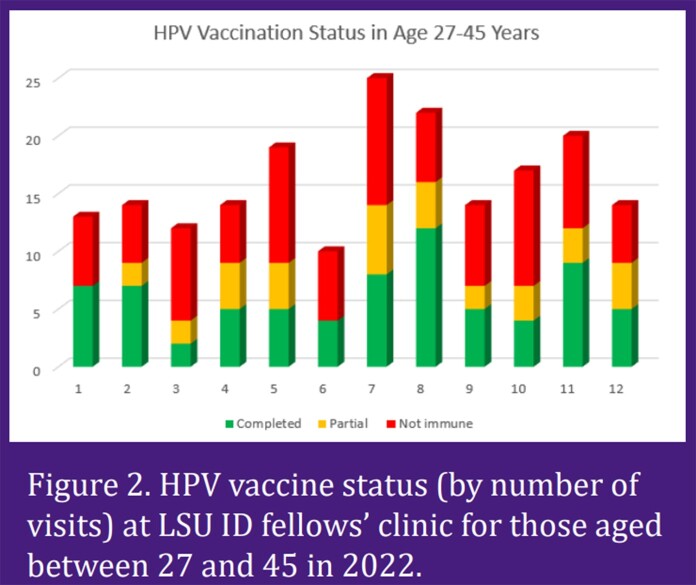

**Conclusion:**

Vaccination compliance among the LSU ID fellows was less than ideal, only 71% of visits for patient aged up to 27 years attending LSU ID fellows’ clinic had completed HPV vaccine series, compared to the Health People 2020 recommendation of 80%. Only 38% of visits for patients aged 27 through 45 years are fully vaccinated with HPV vaccines. Patients in this age group are often labeled “aged out” for HPV vaccines by health maintenance reminders however, due to having HIV, they are still eligible and should be vaccinated. These results led to the institution of quality improvement project to address HPV vaccinations. This intervention will focus on educating all HOP providers about the vaccination recommendations and updating the health maintenance care gaps reminder in the electronic medical record.

**Disclosures:**

**All Authors**: No reported disclosures

